# Social, Economic, and Resource Predictors of Variability in Household Air Pollution from Cookstove Emissions

**DOI:** 10.1371/journal.pone.0046381

**Published:** 2012-10-03

**Authors:** Gautam N. Yadama, John Peipert, Manoranjan Sahu, Pratim Biswas, Venkat Dyda

**Affiliations:** 1 George Warren Brown School of Social Work, Washington University in St. Louis, St. Louis, Missouri, United States of America; 2 Division of General Medical Sciences, Washington University School of Medicine, Washington University in St. Louis, St. Louis, Missouri, United States of America; 3 Advanced Energy Technology Initiative, University of Illinois at Urbana-Champaign, Urbana-Champaign, Illinois, United States of America; 4 Aerosol and Air Quality Research Laboratory, Department of Energy, Environmental and Chemical Engineering, Washington University in St. Louis, St. Louis, Missouri, United States of America; 5 Foundation for Ecological Security, Papagni Regional Cell, Madanapalle, India; University of Maribor, Slovenia

## Abstract

We examine if social and economic factors, fuelwood availability, market and media access are associated with owning a modified stove and variation in household emissions from biomass combustion, a significant environmental and health concern in rural India. We analyze cross-sectional household socio-economic data, and PM_2.5_ and particulate surface area concentration in household emissions from cookstoves (n = 100). This data set combines household social and economic variables with particle emissions indexes associated with the household stove. The data are from the Foundation for Ecological Society, India, from a field study of household emissions. In our analysis, we find that less access to ready and free fuelwood and higher wealth are associated with owning a replacement/modified stove. We also find that additional kitchen ventilation is associated with a 12% reduction in particulate emissions concentration (p<0.05), after we account for the type of stove used. We did not find a significant association between replacement/modified stove on household emissions when controlling for additional ventilation. Higher wealth and education are associated with having additional ventilation. Social caste, market and media access did not have any effect on the presence of replacement or modified stoves or additional ventilation. While the data available to us does not allow an examination of direct health outcomes from emissions variations, adverse environmental and health impacts of toxic household emissions are well established elsewhere in the literature. The value of this study is in its further examination of the role of social and economic factors and available fuelwood from commons in type of stove use, and additional ventilation, and their effect on household emissions. These associations are important since the two direct routes to improving household air quality among the poor are stove type and better ventilation.

## Introduction

Around the globe, 2.7 billion people depend on traditional biomass fuels to meet their daily household energy needs for cooking and heating, and the estimates are for this number is to rise to 2.8 billion by 2030 [1]. Burning wood, crop waste, grasses, shrubs, and dung is inefficient, unhealthy, and has adverse effects on the environment. Mounting interest from governments and international multilateral agencies in sustainably replacing traditional cookstoves with improved stoves and substitute cleaner fuels for solid biomass is motivated by the potential to improve human health and local environments as well as climate benefits [2].

Approximately 2 million people die annually because of indoor air pollution from solid biomass combustion, and 99 percent of these deaths occur in developing countries [3], with 570,000 annual deaths in India [4], [5]. Adverse health conditions associated with exposure to biomass emissions include: chronic bronchitis [6]; chronic obstructive pulmonary disease (COPD) and asthma [6], [7], acute respiratory infections [7], [8]; decreased lung function [9]; tuberculosis [10], nasopharyngeal, laryngeal, and lung cancer [11], pneumonia [12], and low birth weight among children [13]. Recent studies suggest that biomass combustion is an even greater risk factor for COPD than cigarette smoking, particularly in India where 156 million households still depend on solid biomass for cooking and heating [14]. The urgency to address the health of millions is reflected in the newly formed Global Alliance for Clean Cookstoves to promote improved biomass cookstoves [15]. Replacement cookstoves designed for high efficiency and low emissions, and modifications to ventilation in the cooking area, are two solutions to reducing exposure to harmful household air pollution. The available evidence suggests that a household’s behavioral response to interventions depends on livelihood strategies, household characteristics, variability in solid biomass availability, culture-based preferences around food preparation, and the cost of obtaining traditional fuels [16], [17]. There is some initial evidence that when traditional fuels are abundant, households are less likely to adopt new and cleaner energy solutions [18].

Social and economic class of a household is a significant factor in the type of fuel used, and how efficiently that fuel is combusted. The type of fuel and efficiency of combustion determine the subsequent harmful impacts on the household. There has been greater attention to understanding health outcomes across social and economic groups [19]–[21], including a focus on how access to type of fuel and exposure to varying environmental conditions differ by social and economic class and drive variations in health [22]. The poor primarily use polluting fuels like wood, crop-waste, and dung cakes obtained from common lands – pastures and forests – that are not privately owned and accessible to all members of a community for resource extraction [23]. The dependence of poor on such freely available solid fuels will more likely expose them to higher levels of emissions.

Although many studies have analyzed household cookstove emissions in rural India and throughout the developing world, few examine how social and economic and other contextual factors, such as access to fuelwood from commons, access to markets place households at a continued risk of using traditional stoves and solid biomass fuels [2]. One study that directly analyzes the relationship between socio-economic variables and emissions levels by Dasgupta, et al. concludes that households with higher income and education have lower levels of PM_10_ exposure [24]. The study further investigates factors associated with socio-economic status that affect the level of emissions exposure of household members, including stove type and ventilation. A systematic review of stove research concludes that there is evidence for positive income and education effect on uptake of cleaner stoves and fuels, but it is not across the board, and the need for more evidence on an expanded set of context variables such as fuelwood availability and proximity to markets in addition to income and education on stoves and household air pollution [2].

This paper is in part a response to such calls with a particular focus on testing if social and economic factors, open access to biomass fuel, easy access of village to markets, and media exposure affect the type of household stove used and likelihood of additional ventilation, and their relationship to level of emissions in a household. A particular focus on these factors is especially timely given that India is poised to launch a new, large-scale program – the initiative on improved biomass stoves – in which millions of stoves will be disseminated with the objective of reducing household air pollution [25]. Understanding how socio-economic factors, and access to fuelwood, markets, and media are related to the likelihood of a traditional or an improved stove, and associated levels of cookstove emissions will help in identifying potential barriers to reducing harmful household emissions in biomass dependent rural households [25].

We analyze the importance of these factors on household air quality in rural Andhra Pradesh and Karnataka, India. Particulate emissions indices described in Sahu et al have been calculated for each household and combined with data on social, market access, media exposure, and fuelwood availability from commons to examine variation in emissions levels across households as a function of these variables [26]. The National Institutes of Health has underscored the need for better understanding of such contextual factors on household air pollution from cookstoves in addition to more research on health risks associated with such emissions [27]. The data available to us do not provide health information and therefore is not possible to associate variations in emissions to specific household health outcomes. In this paper, however, we offer additional empirical evidence for understanding household air pollution, a significant factor in adverse health of the poor, from household variability in social and economic privilege, fuelwood access, market access, and media exposure.

## Methods

Data come from a cross-sectional study of a random sample of households by the energy team of the Foundation for Ecological Security (FES), India. We have obtained a formal approval for this study from Washington University Human Research Protection Office (HRPO) and they have determined that it does not involve activities that are subject to Institutional Review Board oversight. The data obtained for this analysis are anonymized and therefore this activity is not considered to meet federal definitions under the jurisdiction of an IRB and therefore falls outside the purview of the HRPO.

From February 2007 to March 2008, FES installed Deenabhandu dome shaped biogas units in 400 households across 63 out of a total 195 habitations of Thambalapalle-Kalicherla cluster of Andhra Pradesh where they work. Similarly, FES installed Sarala model *chulas* (Hindi for cookstoves) from November 2005 to December 2008 in 1066 households across 45 habitations out of a total 68 habitations where they work in the adjacent contiguous region of Rayalpadu, Karnataka. The replaced stoves in this study were approximately between 18 and 30 months old at the time of data collection by FES. FES is engaged in these regions and these habitations in a variety of ecological restoration projects including the implementation of more efficient cookstoves.

Households were selected through a stratified random sampling of habitations from among all the habitations where a proportion of the traditional stoves were replaced with new stoves in these two regions. Households within the selected habitations were randomly chosen. Thirty habitations were randomly selected from these clusters of habitations –10 habitations each from Thambalapalle and Kalicherla region of Andhra Pradesh, and another 10 habitations from Rayalpadu region of Karnataka. In all these 30 habitations, there are households that received improved stoves and those with traditional stoves. In each habitation, four households were selected for a total sample size of 120 households. After excluding cases with missing data, our analysis in this paper is based on 100 households with traditional and replacement stoves. Data obtained for this analysis does not contain identifiable information on households and the villages.

Social-economic data on age of respondent, caste, wealth, livelihood strategies, availability of commons for biomass, perceptions of fuelwood scarcity, whether household owns a TV (proxy for media exposure), presence of an all weather road access to the village (proxy for market access), and household air quality are available for each household in this data (Table 1). Emissions sampling was conducted concurrently with household surveys, wherein PM_2.5_ concentration and particulate surface area concentration were measured for cookstove emissions in these households. PM_2.5_ concentration data were gathered using a personal aerosol monitor – the TSI SidePak AM 510, St. Paul, MN, USA – and a UCB monitor (designed at University of California Berkeley). The real-time surface area concentration of airborne particles deposited in the tracheobronchial and alveolar regions of the lung were collected using a nanoparticle surface area monitor, the TSI AEROTRAK 9000, St. Paul, MN, USA. PM_2.5_ and particulate surface area were measured for households with traditional biomass stoves, replacement Deenabandhu model biogas stoves, and Sarala model improved chulas with flue and chimney. Particles deposited in tracheobronchial (TB) and alveolar (A) regions of the lung were calculated using the established deposition curves given by International Commission on Radiological Protection (ICRP). Then the surface area size distributions obtained from different stoves were weighted with the deposition fraction (that depends on particle properties) is integrated for the desired size range of particles for determining the SA of particles deposited in lung.

**Table 1:** Sample Characteristics of Social, Economic, and Emissions Variables.

Variable	%
Household Owns Replacement/Modified Stove	
No	49
Yes	51
Household Has Ventilation in Kitchen	
No	57
Yes	43
Level of Caste Privilege	
Extremely Underprivileged	27
Underprivileged	32
Privileged	41
Quantity of Common Land Available	
≤400 hectares	50
>400 hectares	50
Household Perceives Fuelwood Scarcity	
No	21
Yes	79
Household Owns TV	
No	67
Yes	33
There Is an All-Weather to the Household’s Village	
No	38
Yes	62
	**Mean (SD)**
Particle Index – Tracheobronchial	0.16 (0.16)
Particle Index - Alveolar	0.15 (0.16)
Age of Respondent (Years)	41.07 (13.64)
Livestock Index	3.21 (3.93)
Land Owned in Hectares	1.30 (1.43)
Number of School Years for Household Head	2.76 (4.30)

Emissions concentrations were sampled at two distances from the stoves: Location 1– breathing-zone of the stove user (within 0.5 m away); Location 2– distances representing where non-stove-using members would carry out daily activities (between 1–5 m away). We use only the Location 1 emissions data in our analysis. A detailed description of the IAQ sampling methods and development of emissions indices is provided in a previous publication by Sahu et al [26]. Sahu et al focus exclusively on the utility of an emissions index for particles lodging in the tracheobronchial and alveolar regions, and provide methodological details for calculating such an index.

### Emissions Indices Calculation

The emissions indices were calculated based on the measured emission values normalized to the range between safety standard and lowest emission level observed. The dose metric for which no established safety standard is available, the highest value observed during the field measurements and/or the upper limit of the instrument was used for calculating the index. The index calculations are formulated as described in Sahu et al [26] as,
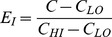



Where, 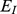
is the emissions index corresponding to the dose metric selected, C is the Surface area concentration (in tracheobronchial or alveolar region) in µm^2^/cm^3^ for calculating SA index at TB and A region. C_LO_ is the lowest concentration and C_HI_ is taken as the highest concentration. The normalized indices values ranged between 0 to1, where “0” indicates the lowest emissions and “1” indicates highest emissions. This indices approach was used for comparison of emissions levels from all the cookstoves studied in the field campaign. More details about the types of stove and detailed explanation can be found from Sahu et al., as we provide only a brief summary of the methodology for calculating the indices used in our analysis. The primary objective of this analysis is to use the emissions indices as outcome measures and examine the key social, household, livelihood and other factors related to household emissions harmful to human health.

### Measures and Statistical Analysis

Social and economic privilege is status and associated benefits to a household that directly flow from caste and wealth. The effect of social and economic privilege on emissions is through the type of stove or fuel a household uses. As households are socially advantaged and economically secure they are able to afford stoves that are efficient and even cleaner fuels. In addition, privilege conferred by income or social caste increase household interaction with the outside world, and the flow of new information, and may increase the chances of adopting a new stove. Therefore, we first examined the relationship between social and economic variables and the type of stove used, and the presence of additional ventilation in the household. We then tested the relationship between stoves used, additional ventilation, and the level of particulate concentration in the household. In doing so, we sought to understand the associations between social and economic privilege and household air pollution, harmful to the environment and human health. We used Stata Version 10 for our statistical analysis.

We first fitted a multivariable logistic regression model using stepwise selection to examine associations between social and economic privilege of a household and owning a replacement/modified cookstove - a stove with a flue or chimney to vent smoke or a biogas unit that eliminates smoke. Traditional stoves used by households include biomass stoves: 3-stone construction stoves, or earthen *chulhas* without a chimney; and kerosene stoves. The predictors of owning an improved stove include the respondent’s age, household caste, the quantity of land owned by the household in hectares, livestock ownership, the years of education of household head, quantity of common land available for fuelwood collection, whether the household perceives fuelwood scarcity, whether the household owns a television, and if there is an all-weather road to the households’ village, a proxy for market access.

In this survey, the respondent was the household head. We hypothesized that older household heads, more constrained by social norms, may have a more difficult time adopting new, or modifying extant cooking technology or ventilation. In addition, older household heads may be less influenced by media to shift to new technologies. Household caste variable was coded into four categories of caste privilege based on consultation with FES about local norms in the study villages. We relied on local experts’ classification of castes and the privilege conferred from belonging to these categories: (1) highly privileged; (2) somewhat privileged; (3) under-privileged; and (4) extremely underprivileged. In our analysis, the highly privileged and somewhat privileged groups were collapsed into a single category, yielding a three-category variable. We hypothesized that privileged caste households would be more likely to have a replacement stove and additional ventilation.

Quantity of land owned was defined in hectares of irrigated and non-irrigated land owned by the household, a proxy for household wealth. We log-transformed to obtain a normal distribution of land-owned variable. Livestock-ownership index weights small livestock (sheep and goats) at 0.1 and large livestock (cows, buffalo, pigs, others) at 1.0, also a proxy for household wealth [23]. We hypothesized that households with more land and greater livestock wealth would be more likely to have a replacement stove and additional ventilation. The quantity of common land available to households for collecting fuelwood was used as a proxy for availability of free or low cost biomass fuel. Freely available fuelwood lowers the opportunity costs to shifting to newer stoves and is an important disincentive to adopt replacement stoves. Access to more common land equates to greater availability of free fuelwood, where 1 is greater than 400 hectares of common land available, and 0 is 400 or less hectares of common land available. Along with the quantity of common land available for fuelwood collection, perception of fuelwood scarcity tracks the pressure households may feel to adopt a replacement stove or modify their own stove to improve combustion efficiency. We hypothesized households with access to less common land (thereby less free fuelwood) and households perceiving greater fuelwood scarcity would be more likely to have a replacement stove.

Television ownership tracks the degree to which households might be exposed to media or external information disseminating better cookstove technologies which have been shown to impact household energy decisions [28]. We hypothesized that households owning a television would be more likely to have a replacement stove and have additional ventilation. All weather roads allow for easier and more travel into and out of the household’s village. All weather roads increase market penetration, but also of government programs, and overall increase bi-directional interaction between households and outside influences [29], [30], [31]. Travelling out of the village may enable household members more easy access to urban and peri-urban centers where new cooking technologies are available. Further, if a village has an all-weather road, extension workers from NGOs and other groups disseminating emissions-reducing cooking technology and information will have an easier time reaching the village regularly, raising awareness about harmful stove emissions, and the benefits of new stoves. Therefore, we hypothesized that households in a village with an all-weather road would be more likely to have a replacement stove and have additional ventilation.

Previous studies have also shown that variation in indoor emissions could be due to home ventilation unrelated to a stove [32], [33]. Therefore, we fitted a multivariable logistic regression model using stepwise selection to estimate the effect of socioeconomic privilege on the likelihood of having additional ventilation; whether or not a household has additional ventilation in the kitchen, or room where the stove is predominantly used. In this model we used the same predictors as the model for having a replacement stove. We extended our logistic regression analyses by estimating the predicted probability of having a replacement/modified cookstove for specific categories of households classified by their socioeconomic privilege conferred by education, livestock wealth index, and access to free fuelwood from local common lands. The fit and performance of the logistic regression models was assessed using the likelihood ratio χ^2^ and c statistic.

We then in an Ordinary Least Squares (OLS) regression analysis compared the relative impact of replacement/modified cookstoves and additional ventilation on the two types of particulate emission indices from cooking that are harmful to the environment and respiratory health. The first index combines measures of the mass concentration (PM_2.5_) of particulate matter in cookstove emissions with the surface area concentration of particulate emissions deposited in the tracheobronchial (TB) region of the human lung (TB particle index). The second index combines measures of the mass concentration (PM_2.5_) of cookstove emissions with the surface area concentration of particulate emissions deposited in the alveolar (A) region of the human lung (A particle index). For both indices, scores range between 0 and 1, and higher scores represent higher particulate emissions and potential harm to health. The model F-test and the model R^2^ are used to assess the fit and performance of the OLS regression models.

## Results

### Likelihood of Owning a Replacement/modified Cookstove

The likelihood of owning a replacement/modified cookstove increased with livestock wealth and decreased as a household had greater access to biomass from the commons such as forests and other land. The logistic regression model predicting ownership of a replacement/modified cookstove fit the data well: likelihood ratio χ^2^ (2, n = 100) = 18.6, *p*<0.0001; c-statistic  = 0.73 (Table 2). The livestock index, a proxy for wealth, was significantly associated with owning a replacement/modified stove; the odds of owning a replacement/modified cookstove increased with wealth as measured by the number of livestock owned (OR = 1.10, 95% CI: 1.01, 1.21). The quantity of common land available for fuelwood collection was also significantly associated with owning a replacement/modified stove (OR = 0.20, 95% CI: 0.07, 0.85). The odds of a household owning a replacement/modified cookstove were 80% lower when a household had access to commons and therefore more fuelwood. Perceptions of fuelwood scarcity, access to all weather roads, ownership of a TV, age of respondent, the number of school years for household head, the quantity of land owned by the household, and caste of a household did not have a significant effect on owning a replacement/modified stove.

**Table 2:** Adjusted odds ratios for household and village-level variables’ association with having replacement/modified stoves and additional ventilation^a^.

	Replacement/Modified Stove	Additional Ventilation
Variable	Adjusted Odds Ratio (95% CI)	Adjusted Odds Ratio (95% CI)
Level of Caste Privilege	n.s.^b^	n.s.
Log of Land Owned (ha)	n.s.	3.35 (1.74, 6.44)***
Log of Livestock Index	1.10 (1.01, 1.21)*	n.s.
Age of Respondent	n.s.	n.s.
Household Perceives Fuelwood Scarcity	n.s.	n.s.
Household Owns TV	n.s.	n.s.
Number of School Years for Household Head	n.s.	1.14 (1.02, 1.27)*
Quantity of Common Land Available (1 = >400 ha; 0 = ≤400 ha)	0.20 (0.07, 0.85)**	n.s.
There Is an All-Weather to the Household’s Village	n.s.	n.s.

a *NOTE*: CI  =  Confidence Interval; ^b^n.s. denotes a non-significant association that was removed from the model in the stepwise selection process.

* *p*<0.05;

** *p*<0.01;

*** *p*<0.001.

**Figure 1:**
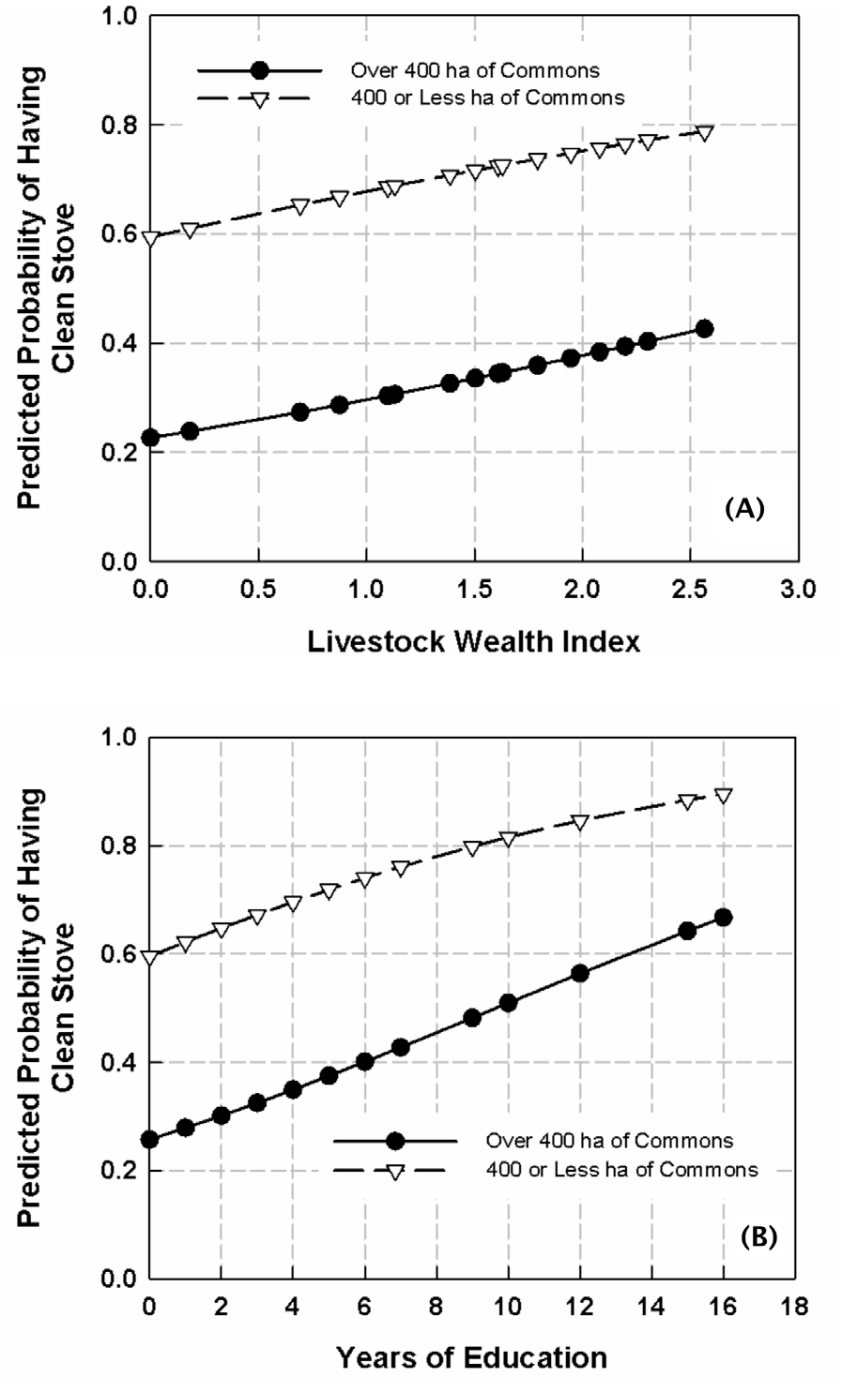
Predicted Probability of Having a Clean Stove by Livestock Wealth and Access to Fuelwood from Commons (A) and by Years of Education and Access to Fuelwood from Commons (B).

We then used the SPost utilities for logistic regression in Stata [34] to compare the predicted probability of owning a replacement/modified stove for households that had access to greater than 400 ha of common land to collect fuelwood to those with less than 400 ha or less of common land across various levels of education and wealth (See Figure 1). Two trends were clear in the predicted probabilities of owning a replacement/modified stove across different levels of livestock wealth and years of education. Among all households, as wealth and education increased, so did the predicted probability of owning a replacement/modified stove. Yet, even as this positive relationship between education and wealth with the likelihood of owning a replacement/modified stove held, those households with greater access to common lands, therefore more free fuelwood and less fuelwood scarcity had a consistently lower predicted probability of owning a replacement/modified stove than households with less access to commons. This finding underscores how, irrespective of caste and wealth privileges, a households’ access to free fuelwood has a bearing on the decision to replace traditional stoves with new stoves.

### Likelihood of Additional Ventilation in the Kitchen

The logistic regression model predicting additional ventilation had a good fit to the data: likelihood ratio χ^2^ (2, n = 100) = 23.0, *p*<0.0001); c statistic  = 0.77 (Table 2). Years of education of household head was significantly associated with having additional ventilation in the kitchen (OR = 1.14, 95% CI: 1.02, 1.27); the odds of a household having additional ventilation increased 14% for each additional year of education of household head. Wealth, as in land owned, was also significantly associated with having additional ventilation: the odds of having additional ventilation increased with amount of land owned (OR = 3.35, 95% CI: 1.74, 6.44).

### Effect of Owning a Replacement/modified Cookstove and Having Additional Ventilation on Household Emissions

Predictors of particle emissions from household cookstoves, our normative outcome measure of household emissions, were estimated using OLS regressions. The effect on household emissions from owning a replacement/modified cookstove and additional household ventilation are shown in Table 3 (Models 1–3). Emissions were differentiated by their deposition in the tracheobronchial region (TB particle index) and alveolar region (A particle index). Additional ventilation in a home significantly reduced emissions of the TB particle index (Table 3– Models 1–3). Unadjusted, both having a replacement/modified stove and additional ventilation were significantly associated with lower TB particle index scores (Tables 3, Models 1 & 2). Having a replacement/modified stove, however, was not significantly associated with the TB particle index after controlling for additional ventilation in the kitchen (Table 3 - Model 3). Additional ventilation, after controlling for ownership of a replacement/modified stove, was associated with a 12% reduction in the TB particle index in a household. In the unadjusted regression (Table 3 - Model 4), additional ventilation was significantly associated with our second emissions index – A particle index. Multivariable regression analysis, however, indicated that neither ventilation nor ownership of a replacement/modified stove were significantly associated with this second particulate index when controlling for the other (Table 3– Model 6).

**Table 3:** Predictors of household particle emissions^a^.

	Tracheobronchial particle emissions index			Alveolar particle emissions index		
	Model 1	Model 2	Model 3	Model 4	Model 5	Model 6
Household Has Ventilation in the Kitchen	−0.14** (0.04)	–	−0.12* (0.04)	−0.09* (0.05)	–	−0.05 (0.05)
Household Owns Replacement/modified Stove	–	−0.09*(0.04)	−0.02 (0.05)	–	−0.07†(0.04)	−0.02 (0.05)

a OLS regression b coefficient (robust standard error).

* *p*<0.05;

** *p*<0.01;

† Trended toward significance at *p*<0.10.

## Discussion

Our results indicate that the less wealthy households are exposed to higher concentrations of particulate emissions. Better-off households were more likely to have additional ventilation and having additional ventilation effectively reduced particulate emissions concentrations. Even among these rural households, the well-to-do modified their homes to improve ventilation and potentially offset emissions from a poorly functioning stove over time. Notably, reductions in emissions, in this study were not observed from owning a replacement/modified cookstove. Emissions were reduced due to additional ventilation that is unrelated to the stove. One possibility is that the replacement biomass stoves with chimneys may not have reduced particulate emissions concentrations because of flues that functioned sub-optimally or maintenance requirements that were beyond the capability of households. FES teams in their routine community visits have observed several replacement stoves with malfunctioning flues. Informal interviews with such households by FES village teams indicate that household members were unable to perform regular maintenance which resulted in broken flues. Perhaps the level of maintenance required for the stoves to work properly was beyond the capability of a household. Our findings resonate with research indicating high maintenance requirements seem unreasonable to poor rural households, leading to disrepair of replacement stoves [32], [35]. Stoves, implemented by FES, at the time of study, were approximately 30 months old, underscoring the need for maintenance and support after installation as an important factor for consideration in improving household air quality. More nuanced research is warranted to understand how rural households address household air quality through better maintenance and proper functioning of stoves or through such other measures as improved ventilation.

Another insight from this study that warrants greater attention in future studies is the reduced propensity to shift to newer stoves when households have greater access to free fuelwood from the commons, as measured by amount of commons available to a household. Ours is one of the few studies that examine the association between fuelwood availability and reduced likelihood of shifting to new stoves. While we are cautious about this conclusion given that our analysis is from cross-sectional data, this association between abundant fuelwood from commons and incentives to shift to new energy systems needs further study to test for a causal connection.

We conjecture two important pathways by which biomass access from commons play a significant role in the uptake and sustained use of replacement cookstoves. First, sheer availability of fuelwood, irrespective of quality, could significantly reduce the opportunity cost of shifting to replacement cookstoves. Second, as burden of fuelwood collection from the commons typically falls on women and children, it could be a significant barrier for shifting to newer efficient stoves. As households undervalue women and children’s labor, the perceived opportunity cost of collecting freely available biomass from commons to meet daily energy needs remains low, and therefore potential economic and health gains from shifting to either a biogas stove or cleaner burning woodstove are possibly discounted by poor households. Social caste, age of household head, perceptions of fuelwood scarcity, media and market access were all not significant in having a replacement stove or additional ventilation.

Our analysis points to some important associations, but is also limited. First, our analysis is from cross-sectional data, and we must caution against strict causal attributions. While such data may yield useful insights on an understudied issue, generalizability is less than if we used data with a much larger sample of households representing greater regional variation. While on one hand, our narrow geographical focus controls for household socio-economic and geographical factors that may differ across regions of India, a larger-n study exploring similar research questions that would be generalizable to larger geographical regions may yield a higher impact on future household air pollution reduction interventions. Second, data from a large, randomized control trial directly comparing the effectiveness of replacement/modified cookstoves to traditional stoves and ventilation in reducing indoor emissions, isolating the effect of each intervention have much greater purchase in testing the impact of new stoves. Such randomized control studies are now being implemented in India, and the results from this analysis are useful in providing insights into productive hypotheses for testing such studies. Third, models predicting exposure to cookstove emissions due to socio-economic variables are under-developed in the scientific literature, and, therefore, our models will likely suffer from a degree of inaccuracy. More research including such variables will contribute to a better understanding of the predictors of exposure to cookstove emissions, refining theory, generating more parsimonious models, and yielding evidence that is useful to the design of household air pollution in the field. Our results and analysis in this paper should be viewed in light of these limitations but also as evidence for some robust associations that warrant pursuit in both large sample household surveys and randomized control trials examining sustainability of improved stoves in rural India. Our findings while insightful are associational in nature, and therefore merit further research to establish causal pathways to improved household air quality and health outcomes.

Finally, as we stated previously, the relationship between the emissions indices and actual health outcomes are not available in this study. However, the emissions indices are useful presently as indicators of health impact in as much as they: 1) indicate the scale of particle deposition in two areas of the human lung; 2) can be associated with the PM_2.5_ and surface area concentrations reported in our previous work [26]. We have now designed a randomized control trial in rural India to examine the impact of improved stoves on respiratory health outcomes of the poor, and it will be a further test of the emission indices we used in this analysis.

Despite these limitations, our analysis yields important lessons for the next generation of cookstove programs. While exposure to emissions from cookstoves is presently a rural reality, social and economic inequalities are an important dimension of exposure variation. Variations in exposure to household emissions are likely to increase from new efforts to disseminate clean fuel and stove technologies that are without regard to who is likely to accept and use them in a sustained manner. Our results resonate with findings of a recent systematic review of cookstove studies that social and economic variables are important in the uptake of clean stoves and fuels. We also respond to their call to examine understudied contextual factors such as social marginalization, market access, abundance of fuelwood, possibly risking uptake and sustained use of newer stoves and cleaner fuels [2]. While our results do not show any association between market and media access on uptake of modified stoves, there is a need for sharper focus on barriers that the poorest rural households face in adopting and sustaining replacement cookstoves is urgent. Greater attention towards inclusion of the poorest households is paramount, as findings in our paper suggest that socio-economic and resource conditions are closely coupled with household behavior around stove adoption and use, which in turn determines exposure to household air pollution and subsequent health outcomes [36].
